# Sulfated Lactosyl Archaeol Archaeosomes Synergize with Poly(I:C) to Enhance the Immunogenicity and Efficacy of a Synthetic Long Peptide-Based Vaccine in a Melanoma Tumor Model

**DOI:** 10.3390/pharmaceutics13020257

**Published:** 2021-02-12

**Authors:** Bassel Akache, Gerard Agbayani, Felicity C. Stark, Yimei Jia, Renu Dudani, Blair A. Harrison, Lise Deschatelets, Vandana Chandan, Edmond Lam, Usha D. Hemraz, Sophie Régnier, Lakshmi Krishnan, Michael J. McCluskie

**Affiliations:** 1 Human Health Therapeutics, National Research Council Canada, Ottawa, ON K1A 0R6, Canada; bassel.akache@nrc-cnrc.gc.ca (B.A.); Gerard.agbayani@nrc-cnrc.gc.ca (G.A.); felicity.stark@nrc-cnrc.gc.ca (F.C.S.); yimei.jia@nrc-cnrc.gc.ca (Y.J.); Renu.dudani@nrc-cnrc.gc.ca (R.D.); blair.harrison@nrc-cnrc.gc.ca (B.A.H.); lise.deschatelets@nrc-cnrc.gc.ca (L.D.); vandana.chandan@nrc-cnrc.gc.ca (V.C.); lakshmi.krishnan@nrc-cnrc.gc.ca (L.K.); 2 Aquatic and Crop Resource Development, National Research Council Canada, Montreal, QC H4P 2R2, Canada; Edmond.Lam@nrc-cnrc.gc.ca (E.L.); usha.hemraz@nrc-cnrc.gc.ca (U.D.H.); sophie.regnier@nrc-cnrc.gc.ca (S.R.)

**Keywords:** archaeosome, SLA, vaccine, adjuvant, glycolipid, synthetic long peptide, Poly(I:C), cancer

## Abstract

Cancer remains a leading cause of morbidity and mortality worldwide. While novel treatments have improved survival outcomes for some patients, new treatment modalities/platforms are needed to combat a wider variety of tumor types. Cancer vaccines harness the power of the immune system to generate targeted tumor-specific immune responses. Liposomes composed of glycolipids derived from archaea (i.e., archaeosomes) have been shown to be potent adjuvants, inducing robust, long-lasting humoral and cell-mediated immune responses to a variety of antigens. Herein, we evaluated the ability of archaeosomes composed of sulfated lactosyl archaeol (SLA), a semi-synthetic archaeal glycolipid, to enhance the immunogenicity of a synthetic long peptide-based vaccine formulation containing the dominant CD8^+^ T cell epitope, SIINFEKL, from the weakly immunogenic model antigen ovalbumin. One advantage of immunizing with long peptides is the ability to include multiple epitopes, for example, the long peptide antigen was also designed to include the immediately adjacent CD4^+^ epitope, TEWTSSNVMEER. SLA archaeosomes were tested alone or in combination with the toll-like receptor 3 (TLR3) agonist Poly(I:C). Overall, SLA archaeosomes synergized strongly with Poly(I:C) to induce robust antigen-specific CD8^+^ T cell responses, which were highly functional in an in vivo cytolytic assay. Furthermore, immunization with this vaccine formulation suppressed tumor growth and extended mouse survival in a mouse melanoma tumor model. Overall, the combination of SLA archaeosomes and Poly(I:C) appears to be a promising adjuvant system when used along with long peptide-based antigens targeting cancer.

## 1. Introduction

The prospects for treating various forms of cancer have been dramatically improved by novel strategies that successfully unleash the immune system to fight and control tumor growth. For example, immune checkpoint inhibitors (e.g., monoclonal antibodies targeting CTLA-4 or PD-1) have been approved for the treatment of many different types of cancer, such as melanoma and non-small cell lung carcinoma [1,2]. This demonstrates that immune cells can attack and kill cancer cells once properly activated, even in the context of solid tumors. Whether used alone or in combination with checkpoint inhibitors, cancer vaccines offer the potential to refine the immune response and direct it specifically to a tumor-associated antigen (TAA), thereby potentially increasing potency and reducing off-target toxicity [3,4].

While prophylactic vaccines rely mainly on the humoral branch of the immune system, efficacious cancer vaccines most likely require the induction of strong T cell responses. As such, the majority of antigen platforms and adjuvants used for currently marketed prophylactic vaccines are not ideal for therapeutic cancer vaccine formulations. Synthetic long peptides (SLPs), generally 20–35 amino acids in length, have been used widely in cancer vaccine strategies and have been evaluated in both preclinical and clinical studies [5,6]. The length of the SLPs allows for inclusion of both CD8^+^ and CD4^+^ specific epitopes. Epitopes can be pre-screened and selected based on multiple factors, including specificity (wild-type vs. mutated protein), potential binding to major histocompatibility complex (MHC) molecules and immunogenicity. This approach is quite compatible with more novel neo-antigen vaccine strategies, where personalized cocktails based on multiple peptides are designed and synthesized based on a particular cancer patient’s mutanome/neo-epitope repertoire and MHC profile [7,8,9]. However, long peptides on their own are poorly immunogenic, with various adjuvants such as the water-in-oil emulsion Montanide, the toll-like receptor 9 (TLR9) agonist CpG, and the TLR3 agonist Poly(I:C) utilized clinically to enhance their immunogenicity [7,10,11]. While these SLP-based vaccine formulations were shown to induce T cells in clinical trials, the level of immunogenicity was generally not sufficient to induce clinically relevant tumor regression [5]. As such, it is important to identify novel strategies capable of boosting SLP immunogenicity.

Archaeosomes are liposomes formed with archaeal-derived lipids which differ chemically from their bacterial/eukaryotic counterparts by containing: 1) an ether linkage between the glycerol backbone and the lipid tails and 2) unique lipid tails called phytanyl chains, which are composed of repeating branched five-carbon units. Archaeosomes have shown strong activity as adjuvants in a number of vaccine studies [12]. A novel semi-synthetic archaeal glycolipid, sulfated lactosyl archaeol (SLA), has been shown to strongly induce both antigen-specific humoral and cellular immune responses when simply admixed with a variety of target antigens in mice [13,14,15]. In addition, an SLA-based formulation encapsulating the class I-restricted epitope from the tumor-associated tyrosinase-related protein-2 (Trp2) antigen induced a strong CD8^+^ T cell and anti-tumor response in vivo [16]. Head-to-head comparative preclinical studies demonstrated that SLA was superior to a number of adjuvant types, including TLR agonists, in inducing antigen-specific T cell responses [17]. In mice, SLA has been shown to increase 1) antigen retention at the injection site, 2) immune cell recruitment, 3) antigen uptake, and 4) cytokine/chemokine expression [17,18,19]. Finally, SLA-based vaccine formulations can induce efficacious antigen-specific anti-tumor responses in a mouse melanoma model [20]. However, to date, archaeosomes have not been evaluated using long peptides, and therefore it was of interest to determine their utility in this setting. Herein, we evaluated the adjuvant activity of our novel admixed SLA archaeosome formulation in the context of a weakly immunogenic SLP antigen containing the main CD8^+^ T cell epitope from the model antigen ovalbumin (OVA) widely used in vaccine adjuvant studies [21,22]. Poly(I:C), a TLR3 agonist adjuvant routinely used in SLP vaccine formulations [7,23], was included as a comparator. In addition, we also recently found that SLA archaeosomes can synergize with certain TLR agonists (in press), and thus we also tested the adjuvant activity of an SLA archaeosome/Poly(I:C) combination when formulated with the OVA SLP. Finally, we evaluated the ability of the single or the combination adjuvant-based SLP formulations to induce anti-tumor responses when administered therapeutically (i.e., post tumor cell implantation) in an aggressive murine melanoma model. Overall, we show that SLA archaeosomes strongly synergize with Poly(I:C), inducing high levels of antigen-specific CD8^+^ T cells that are efficacious in the context of a therapeutic tumor model. 

## 2. Materials and Methods

### 2.1. Vaccine Preparation and Immunization

Sulfated lactosyl archaeol (SLA; 6’-sulfate-β-D-Galp-(1,4)-β-D-Glcp-(1,1)-archaeol) was synthesized as described previously [24]. Empty archaeosomes were prepared as previously described [13]. Briefly, 30 mg of SLA lipid was dissolved in chloroform/methanol; a thin film was formed after removal of solvent under N_2_ gas with mild heating. A vacuum was applied to ensure total removal of trace solvents. Dried lipids were hydrated in 700 µL of Milli-Q water without protein antigen. Lipid dispersions were shaken for 2–3 h at 40 to 50 °C until completely suspended. Next, a brief sonication was applied at 40 °C in an ultrasonic water bath (Thermo Fisher Scientific, Waltham, MA, USA) for up to 60 min until the desired particle size (~100 nm) was obtained. At this point, the volume of the liposome solution was measured, and an appropriate volume of 10× phosphate-buffered saline (PBS) (Millipore Sigma, Burlington, MA, USA) was added to achieve a final concentration of 1× PBS. The pre-formed empty SLA archaeosomes were diluted to a concentration of ~40 mg/mL and stored at 4 °C until used. 

Poly(I:C) was purchased from InvivoGen (San Diego, CA, USA) and prepared according to manufacturer’s instructions at a concentration of 3 mg/mL and stored at −20 °C until used. The OVA SLP (amino acids 253–277: LEQLESIINFEKLTEWTSSNVMEER) was synthesized by JPT Peptide Technologies GmbH (Berlin, Germany). Based on its theoretical isoelectric point of 4, it was dissolved in slightly alkaline solution (PBS/0.1 M NaOH) at a concentration of 4.4 mg/mL and stored at −20 °C until used. No aggregates were observed upon visual inspection.

The 6–8 week old female C57BL/6 mice were obtained from Charles River Laboratories (Saint-Constant, QC, Canada). On the day of immunization, vaccine formulations were prepared by first mixing the required volume of PBS vehicle and adjuvants (empty pre-formed SLA archaeosomes and/or Poly(I:C)) and briefly vortexing. Thereafter, OVA SLP was added and briefly vortexed. The final concentrations of SLA, Poly(I:C), and OVA long peptide in the injected formulations were 20, 1, and 0.6 mg/mL, respectively. Mice (*n* = 5–10/group) were immunized by intramuscular (i.m.) injection (50 µL) into the left tibialis anterior (T.A.) muscle. Adjuvant (1 mg SLA; 50 µg Poly(I:C)) and antigen (30 µg peptide) dose levels were based on data from previous studies conducted in our laboratory. 

### 2.2. Therapeutic Tumor Challenge Model

B16F0-OVA (expressing plasmid-derived full length OVA) cells were obtained from Dr. Edith Lord (University of Rochester, Rochester, NY, USA) and cultured in R10 media (RPMI containing 10% fetal bovine serum (FBS), 1% penicillin/streptomycin, 1% glutamine, and 55 µM 2-Mercaptoethanol (all from Thermo Fisher Scientific)). Solid tumors were induced with subcutaneous (s.c.) injection of 5 × 10^5^ B16-OVA cells in a volume of 100 µL into the lower dorsal area. Mice were immunized, as described above, 3, 10, and 17 days following tumor challenge. From day 8 onwards, diametrically perpendicular measurements of tumor size (width and length) were measured 2–3 times per week using Digimatic Digital calipers (Mitutoyo 500196, Aurora, IL, USA). An approximation of tumor volume, expressed in mm^3^, was calculated by multiplication of length × width × width/2. Animals were monitored throughout the duration of the study. Mice were euthanized when they achieved one of the following humane endpoints: (1) the tumor volume exceeded 2000 mm^3^, (2) ulcerated bleeding tumor, and (3) mice showed signs of clinical illness (e.g., ruffled fur, very little activity, hunched posture, eyes squeezed shut, very sickly). As this was a therapeutic tumor model, only mice that had measurable tumors (>100 mm^3^) at any timepoint prior to the final vaccination dose were included in the analysis.

### 2.3. ELISpot

The levels of OVA-specific T cells were quantified by ELISpot using a mouse interferon (IFN)-γ kit (Mabtech Inc., Cincinnati, OH, USA) as described [25]. To obtain a sufficient number of lymphocytes for measurement of antigen-specific T cells, spleens were mechanically minced with the frosted ends of two glass slides in R10 media. The splenocyte cell suspension was passed through a 70 µm cell strainer and cell concentrations determined on a Cellometer (Nexcelom, Lawrence, MA, USA). The 4 × 10^5^ cells were stimulated in duplicate with peptides corresponding to the CD8^+^ T cell epitope OVA_257–264_: SIINFEKL or the CD4^+^ T cell epitope OVA_266–277_: TEWTSSNVMEER at a final concentration of 2 µg/mL. Both these epitopes were contained within the long peptide antigen used for immunization. Final volume per well was 0.2 mL. Cells were also incubated without any stimulants to measure background responses. Plates were incubated for ~20 h at 37 °C with 5% CO_2_. Then, the plates were washed and developed according to the manufacturer’s instructions. 3-Amino-9-ethylcarbazole (AEC) substrate (Becton Dickinson, Franklin Lakes, NJ, USA) was used to visualize the spots. Spots were counted using an automated ELISpot plate reader.

### 2.4. In Vivo Cytolytic Activity

Cytolytic (CTL) activity in immunized mice was enumerated as described previously [26]. Briefly, donor spleen-cell suspensions from syngeneic mice were prepared. Cells were split into two aliquots. One aliquot was incubated with 10 μM of the CD8^+^ T cell epitope peptide SIINFEKL (JPT Peptide Technologies GmbH, Berlin, Germany) in R10 media. After 30 min of incubation, the non-peptide containing aliquot was stained with low concentration of Carboxyfluorescein succinimidyl ester (CFSE (0.25) μM; Thermo Fisher Scientific), and the second peptide-pulsed aliquot was stained with 10× CFSE (2.5 μM). CFSE labeling was quenched by adding an equal volume of pure ice-cold FBS to each cell aliquot and incubating on ice for 5 min. The two cell aliquots were each washed and resuspended in Hanks’ Balanced Salt Solution prior to being mixed 1:1 and injected into the retro-orbital plexus (total of 20 × 10^6^ cells in a volume of 0.2 mL per mouse) into previously immunized recipient mice. At ~20 to 22 h after the donor cell transfer, spleens were removed from recipients, single-cell suspensions were prepared, and cells were analyzed by flow cytometry on a BD LSRFortessa™ flow cytometer (Becton Dickinson). The in vivo lysis percentage of peptide-pulsed targets was enumerated according to the equation in reference above. 

### 2.5. Statistical Analysis 

Data were analyzed using GraphPad Prism® (GraphPad Software, San Diego, CA, USA). Statistical significance of the difference between groups was calculated by one-way analysis of variance (ANOVA) followed by post-hoc analysis using Tukey’s (comparison between all groups) multiple comparison test. IFN-γ^+^ spot-forming cell levels by ELISpot were log-transformed prior to statistical analysis. Survival curve analyses were carried out using the Mantel–Cox test. For all analyses, differences were considered to be not significant with *p* > 0.05.

## 3. Results and Discussion

### 3.1. IFN-γ^+^ T Cell Response to OVA SLP Vaccine Formulations in Mice

**Figure 1:**
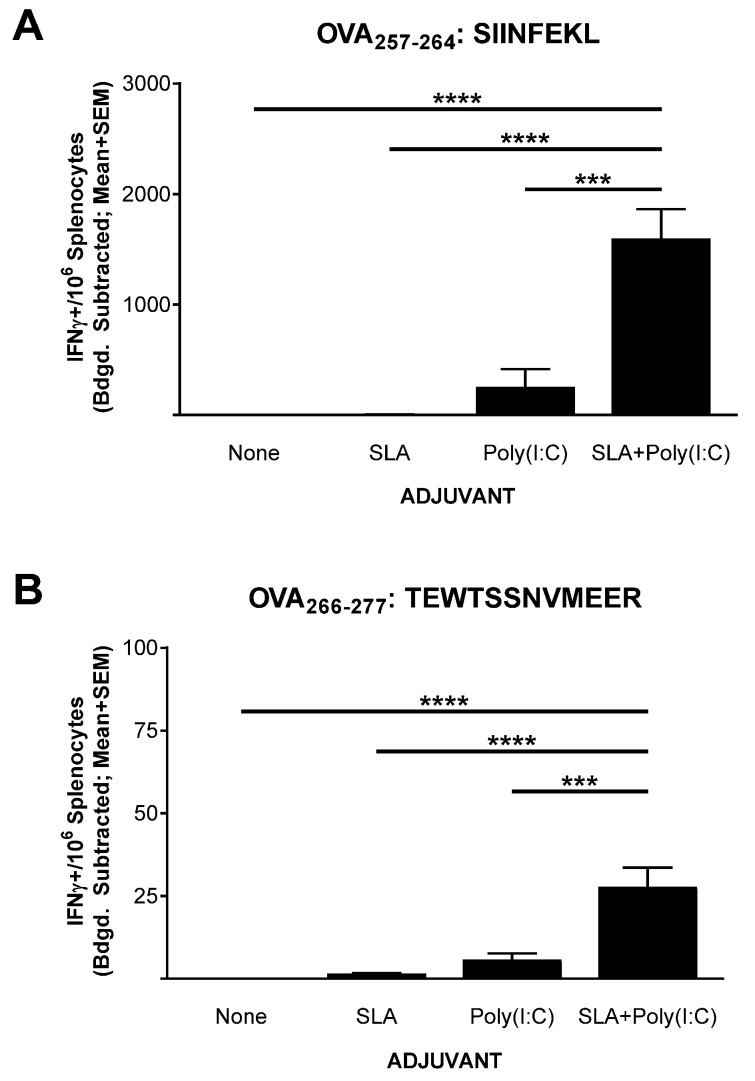
Ovalbumin (OVA)-specific T cells as determined by IFN-γ ELISpot with splenocytes of immunized mice. C57BL/6 mice (*n* = 5/group) were immunized i.m. with OVA synthetic long peptides (SLP) (30 µg) with or without adjuvant on days 0, 7, and 21. Splenocytes were harvested on day 28 (*n* = 5/group) and analyzed by IFN-γ ELISpot when stimulated by OVA CD8^+^ T cell peptide epitope SIINFEKL (IFN: mouse interferon) (**A**) or OVA CD4^+^ T cell peptide epitope TEWTSSNVMEER (**B**). Values obtained with media alone were subtracted from those measured in the presence of the peptides. Grouped data are presented as mean + standard error of mean (SEM). Statistical significance of differences for OVA SLP + sulfated lactosyl archaeol (SLA) + Poly(I:C) vs. other groups is shown: *** *p* < 0.001 and **** *p*< 0.0001 by one-way ANOVA followed by Tukey’s multiple comparisons test.

Mice (*n* = 5 per group) were immunized on days 0, 7, and 21 with OVA SLP alone or in combination with SLA, Poly(I:C), or SLA + Poly (I:C). Splenocytes were collected from the immunized mice on day 28 (7 days post third vaccination) for the assessment of the levels of antigen-specific T cells by IFN-γ ELISpot. Low levels (≤5 IFN-γ^+^ spot forming cells (SFCs)/10^6^ splenocytes) of T cells reactive to the CD8^+^ T cell epitope SIINFEKL were seen in mice immunized with OVA SLP alone or OVA SLP adjuvanted with SLA (Figure 1A). The administration of Poly(I:C) along with OVA SLP resulted in an average ± SEM of 252.5 ± 163.3 IFN-γ^+^ SFC/10^6^ splenocytes reactive to the SIINFEKL CD8^+^ T cell epitope, which was significantly higher than responses seen with OVA SLP alone or OVA SLP + SLA (*p* < 0.0001). The lack of a strong response with SLA alone was somewhat surprising, since we have previously seen that, when formulated with OVA whole protein, SLA archaeosomes generated superior levels of SIINFEKL-specific CD8^+^ T cells compared to a panel of commercial adjuvants, including Poly(I:C) [17]. This indicates that the activity and the hierarchy of adjuvants is quite dependent on the antigen format, with an SLA admixed formulation less capable of inducing OVA-specific CD8^+^ T cells with an OVA SLP antigen than with OVA whole protein. In addition, the nature of the peptide and its physical location in relation to the archaeosome may also have an impact. While an admixed peptide/archaeosome formulation was used in this study, SLA-based archaeosomes have been previously shown to induce T cell responses to an encapsulated Trp2 epitope [16]. Similarly, archaeosomes composed of archaeal total polar lipids were not able to induce protective responses to *Listeria monocytogenes* when a dipalmitoylated 20 a.a.-long lipopeptide antigen targeting listeriolysin was admixed with the archaeosomes [27]. However, efficacious responses were observed when the antigen was encapsulated within the archaeosomes. In addition, other factors such as antigen dose and immunization schedule may impact the ability of an admixed SLA formulation to induce immune responses to long peptide antigens. 

A sharp increase in IFN-γ^+^ SFCs was detected in mice immunized with the SLA + Poly(I:C)-adjuvanted formulation with a mean ± SEM number of IFN-γ^+^ SFC/10^6^ splenocytes of 1597 ± 266.5 in this group. This was > six-fold greater than the levels measured in splenocytes from mice immunized with OVA SLP+ Poly (I:C) (*p* < 0.001). Likewise, when splenocytes were stimulated with the CD4^+^ T cell epitope peptide (TEWTSSNVMEER) found within the sequence of the OVA SLP, low levels of IFN-γ^+^ SFCs (<3 per 10^6^ splenocytes) were measured in splenocytes from mice immunized with vaccine formulations containing peptide alone or OVA SLP + SLA (Figure 1B). In contrast, means ± SEMs of 5.8 ± 1.9 and 27.8 ± 5.8 were measured in splenocytes from mice immunized with OVA SLP + Poly (I:C) and OVA SLP + SLA + Poly (I:C), respectively. While the overall level was lower than that seen with CD8^+^ T cells, the increase in antigen-specific CD4^+^ T cells by the combination adjuvant formulation was statistically higher than all other formulations, including the Poly(I:C)-adjuvanted formulation (p < 0.001). Adjuvant combinations have been used extensively to enhance the immunogenicity of various vaccines [28,29], for example, AS01B™ (a liposome-based vaccine adjuvant system containing the TLR4 agonist 3-O-desacyl-4ʹ-monophosphoryl lipid A (MPLA) and the saponin QS-21) is used in the Shingles vaccine Shingrix™, and AS04™ (a combination of the TLR4 agonist MPLA and aluminum phosphate) is used in the hepatitis B vaccine Fendrix™ [30]. We have recently shown that SLA archaeosomes can synergize with various TLR agonists (e.g., CpG, Poly(I:C)) when used with a whole protein OVA-based vaccine (in press). In those studies, the combination of Poly(I:C) and SLA archaeosomes induced superior antigen-specific CD8+ T cell responses. The results presented herein not only confirm the synergy between these two adjuvants but do so with a poorly immunogenic antigen, namely a long peptide. It would be of interest in future studies to further elucidate the mechanism of action behind the synergy between these different adjuvant types.

### 3.2. Functionality of CD8^+^ T Cell Response to OVA SLP Vaccine Formulations in Mice

**Figure 2:**
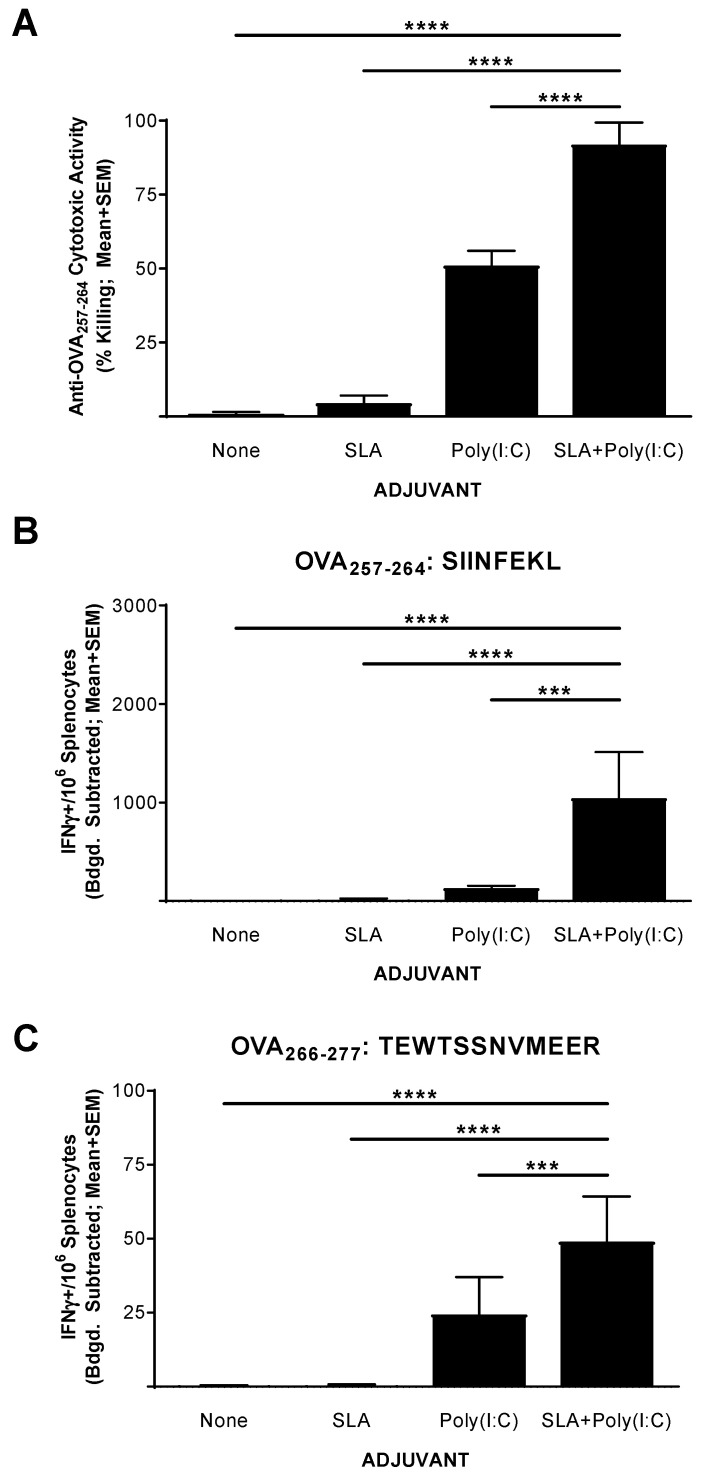
Functionality of OVA-specific T cells as determined by in vivo cytolytic assay in immunized mice. C57BL/6 mice (*n* = 5/group) were immunized i.m. with OVA SLP (30 µg) with or without adjuvant on days 0, 7, and 14. On day 19, mice were administered CFSE-labeled cells that had been pulsed with the OVA CD8^+^ T cell epitope, SIINFEKL. Splenocytes were harvested on day 20 and analyzed for in vivo cytolytic activity (**A**) or by IFN-γ ELISpot when stimulated by OVA CD8^+^ T cell peptide epitope SIINFEKL (CFSE: Carboxyfluorescein succinimidyl ester) (**B**) or OVA CD4^+^ T cell peptide epitope TEWTSSNVMEER (**C**). For ELISpot, values obtained with media alone were subtracted from those measured in the presence of the peptides. Grouped data are presented as mean + standard error of mean (SEM). Statistical significance of differences for OVA SLP + SLA + Poly(I:C) vs. other groups is shown: *** *p* < 0.001 and **** *p* < 0.0001 by one-way ANOVA followed by Tukey’s multiple comparisons test.

The functionality of the CD8^+^ T cell response generated by the above vaccine formulations was confirmed in an in vivo CTL assay. Mice (*n* = 5 per group) were immunized on days 0, 7, and 14 with OVA SLP alone or in combination with SLA, Poly(I:C), or SLA + Poly (I:C). Splenocytes were collected on day 20 (6 days following the third vaccination) and analyzed for in vivo cytolytic activity and by IFN-γ ELISpot. Supporting the results obtained with the IFN-γ ELISpot assay above, the percentage killing of SIINFEKL-pulsed cells in mice administered the unadjuvanted or SLA-adjuvanted vaccine formulations was quite low (i.e., means ± SEMs of 1 ± 0.6 and 4.5 ± 2.6, respectively; Figure 2A). The inclusion of Poly(I:C) in the vaccine formulation resulted in a moderately high level of killing with a mean ± SEM of 51% ± 5, which was significantly higher than seen in mice administered with the unadjuvanted or the SLA-adjuvanted OVA SLP vaccine formulations (*p* < 0.0001). A significantly higher level of SIINFEKL-specific cytolytic activity was seen in the mice immunized with OVA SLP + SLA + Poly (I:C) vs. OVA SLP + Poly (I:C), with a mean ± SEM of 92% ± 3.3 (*p* < 0.0001). The level of killing seen here with the OVA SLP + SLA + Poly(I:C) formulation compares favorably to previous results obtained in mice immunized with OVA whole protein admixed with SLA, where ~75% cytolytic activity was measured using a similar assay [13].

To confirm whether the levels of killing observed were linked to the number of Ag-specific T cells, we also analyzed the splenocytes of these mice using an IFN-γ ELISpot as outlined above. As expected, immunization with OVA SLP + SLA + Poly(I:C) resulted in significantly higher levels of SIINFEKL-reactive CD8^+^ and TEWTSSNVMEER-reactive CD4^+^ IFN-γ^+^ T cells than vaccination with any of the other tested vaccine formulations (Figure 2B,C, *p* < 0.001). Interestingly, the levels of IFN-γ^+^ SFCs when stimulated with SIINFEKL obtained in mice which had received CFSE-labeled cells were lower than those obtained in mice which had not received CFSE-labeled cells (Figure 1). While the dosing schedule was different, this may be partly due to trafficking of SIINFEKL-specific T cells from the spleen to the periphery following intravenous delivery of the target cells for the in vivo CTL assay. 

### 3.3. Anti-Tumor Activity of OVA SLP Vaccine Formulations in a Therapeutic B16-OVA Tumor Challenge Model

**Figure 3:**
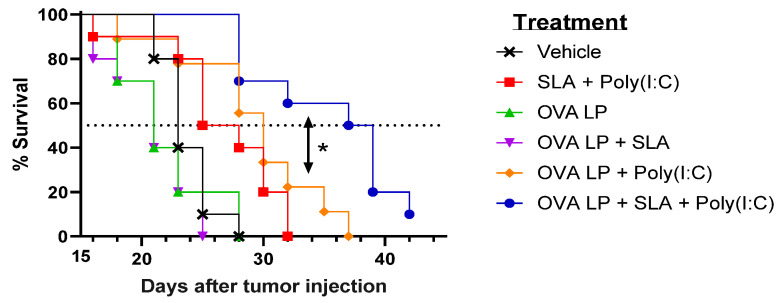
Efficacy of OVA SLP vaccine formulations in a B16-OVA tumor model. C57BL/6 mice (*n* = 10/group) were injected s.c. with 5 × 10^5^ B16-OVA cells on day 0. Following tumor implantation, animals were immunized i.m. with OVA SLP (30 µg) with or without adjuvant on days 3, 10, and 17. Animal survival, clinical signs, and tumor growth were monitored 2–3 times per week. Mice were euthanized once they reached a humane endpoint. Statistical significance of difference for OVA SLP + SLA + Poly(I:C) vs. OVA SLP + Poly(I:C) is shown: * *p* < 0.05 by Mantel–Cox test.

To evaluate the potential benefit of SLA + Poly(I:C) as an adjuvant combination in an SLP vaccine for oncology applications, the various vaccine formulations were also evaluated in the aggressive B16-OVA melanoma tumor model. Mice (*n* = 10/group) were administered vaccines 3, 10, and 17 days following s.c. implantation of B16 melanoma cells engineered to express ovalbumin protein. An adjuvant alone control group received SLA + Poly(I:C) without OVA SLP to determine any potential impact of activation of the innate immune system by the adjuvants on tumor growth. Tumor growth was monitored, and mice were euthanized once they achieved one of the pre-established humane endpoints. There was no significant difference in survival between groups of mice which received vehicle, OVA SLP alone, or OVA SLP + SLA (median survival of 21–23 days; Figure 3 and Table 1). 

**Figure 4:**
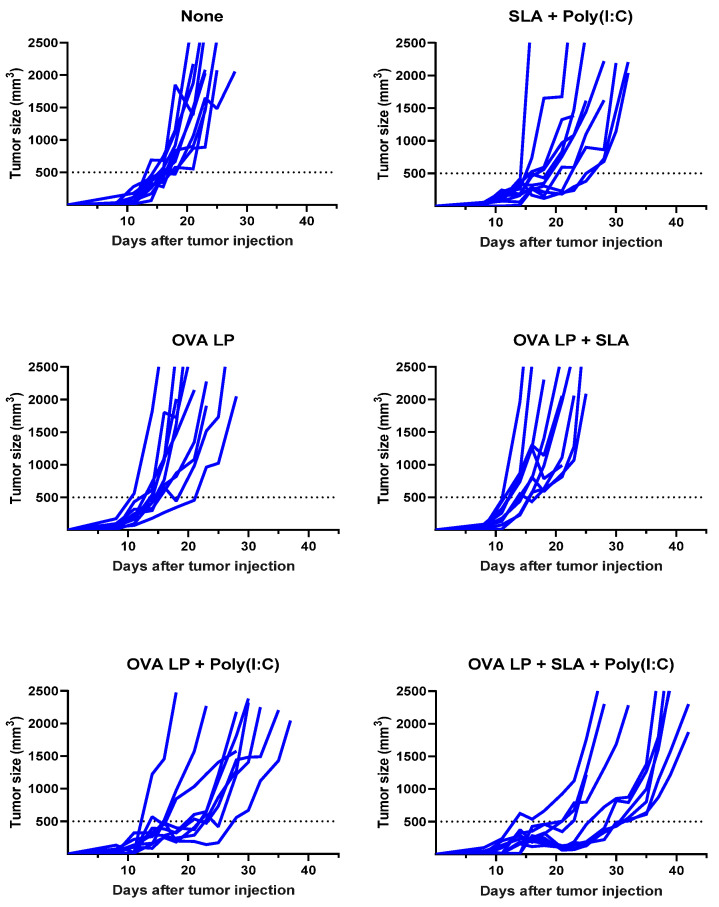
Kinetics of B16-OVA tumor growth in individual mice. C57BL/6 mice (*n* = 10/group) were injected s.c. with 5 × 10^5^ B16-OVA cells on day 0. Following tumor implantation, animals were immunized i.m. with OVA SLP (30 µg) with or without adjuvant on days 3, 10, and 17. Tumor sizes were monitored 2–3 times per week and tumor volumes calculated (length × width × width/2).

Meanwhile, a very modest increase (median survival of 26.5 days; *p* < 0.05 vs. vehicle alone group) was observed following administration of SLA + Poly(I:C) without any antigen. The delay in tumor growth with the adjuvant control was not totally surprising, as Poly(I:C) when administered alone has been shown to activate natural killer cells and suppress tumor growth in a similar B16 melanoma model [31], while SLA has been shown to have inherent immunostimulatory effects [19]. Median survival was further extended in animals which received either of the two formulations shown to generate the strongest cytolytic activity, namely OVA SLP + Poly(I:C) and OVA SLP + SLA + Poly(I:C) (median survival of 30 and 38 days, respectively). The activity of these formulations is further confirmed when directly measuring tumor growth, whereby slowest tumor growth was observed in mice receiving OVA SLP in combination with SLA + Poly(I:C) (Figure 4). The use of SLA + Poly(I:C) as a combination adjuvant with OVA SLP gave significantly longer survival than was obtained with either the adjuvants alone (i.e., SLA + Poly(I:C); *p* < 0.01) or OVA SLP in combination with Poly(I:C) or SLA (*p* < 0.05; Figure 3). Overall, these results suggest that the ability of the SLA + Poly(I:C) adjuvant combination to induce strong CD8^+^ functional T cells translates into slower tumor growth and extended survival in a vigorous murine tumor model and are very encouraging for the future development of SLP cancer vaccines.

The advantages of SLP, which include relative ease/speed of production and ability to specifically target select epitopes, make it an ideal platform for cancer vaccines in general and neo-epitope based strategies in particular. Their potential success in the clinic depends in part on adjuvant formulations capable of enhancing their immunogenicity to generate a sufficiently large pool of tumor-targeting T cells. The SLA + Poly(I:C) adjuvant combination utilized here was especially potent in inducing high levels of both Ag-specific CD8^+^ and CD4^+^ T cells and was markedly superior to Poly(I:C) alone. We still do not fully understand the mechanism of action behind the synergy between SLA and Poly(I:C). In a combination adjuvant screen utilizing whole protein antigens, we saw that the SLA synergized most strongly with nucleic acid-based agonists of intracellular TLRs, namely Poly(I:C) and CpG, but not with the small molecule based TLR7/8 agonist R848 (in press). As SLA is negatively charged, it is unlikely to bind CpG or Poly(I:C) directly. We also found that co-administration of a non-archaeal DPPC/DMPG-based liposome with Poly(I:C) or CpG did not lead to an enhancement in their adjuvant activity with either a whole protein or a long peptide based antigen (data not shown). Further studies will be needed to confirm whether the synergy is due to activation of different inflammatory pathways by these adjuvants or due to some change in their distribution/retention. In addition, studies characterizing the impact of the adjuvant combination on the activity of antigen presenting cells, such as dendritic cells, or on the expression of activation markers on antigen-specific T cells could help explain the synergy observed between these adjuvant types.

Combination adjuvant strategies have been utilized with SLP-based vaccines in the past in an effort to enhance their activity. The inclusion of the TLR9 agonist CPG7909 with the water-in-oil emulsion Montanide™ ISA 51 in peptide-based vaccine formulations has been shown to enhance the levels of antigen-specific T cells clinically, although no clear improvement of disease outcome was reported [32,33,34]. While Montanide-based formulations can initially enhance immune responses, they do form long-lasting antigen depots that can sequester the generated T cells and lead to attenuation of their overall activity over time [35]. The increased interaction between the T cells and the antigen at the immunization site correlated with an increase in apoptosis of the antigen-specific T cells. Hailemichael et al. also demonstrated that shorter-lived vaccine formulations preferentially induced T cell localization to the tumor and increased anti-tumor activity. SLA archaeosomes mediate a short-term depot effect on antigen of <48 h [18] while still stimulating strong and long-lasting cellular responses to a variety of antigens in preclinical models [13,14]. These properties have the potential to make it a more ideal partner for adjuvant combination strategies including TLR agonists. Future studies evaluating the activity of SLA archaeosomes with other TLR agonists and/or SLP targeting self-antigens are planned. In addition, it would be of interest to evaluate the safety and the tolerability of the adjuvant combination formulations in more detail.

## 4. Conclusions

SLA archaeosomes combined with the TLR3 agonist Poly(I:C) comprise a powerful adjuvant system for SLP-based vaccines, inducing high levels of antigen-specific T cells that are functional and efficacious in a therapeutic tumor model. These data support the further development of SLA + Poly(I:C) as an adjuvant formulation for SLP-based vaccines targeting cancer.

## Figures and Tables

**Table 1:** Median survival of B16-OVA challenged mice.

Vaccine Treatment	Median Survival (Days)
Vehicle	23
Sulfated Lactosyl Archaeol (SLA) + Poly (I:C) (No Antigen)	26.5
Ovalbumin synthetic long peptide (OVA SLP)	21
OVA SLP + SLA	21
OVA SLP + Poly (I:C)	30
OVA SLP + SLA + Poly (I:C)	38

## Data Availability

The data presented in this study are available on request from the corresponding author. The data are not publicly available due to privacy concerns.
